# The interplay of SARS-CoV-2 and *Clostridioides difficile* infection

**DOI:** 10.2217/fmb-2020-0275

**Published:** 2021-04-13

**Authors:** Sahil Khanna, Colleen S Kraft

**Affiliations:** ^1^Division of Gastroenterology & Hepatology, Mayo Clinic, Rochester, MN 55905, USA; ^2^Department of Pathology & Laboratory Medicine, Emory University, Atlanta, GA 30322, USA; ^3^Division of Infectious Diseases, Emory University, Atlanta, GA 30322, USA

**Keywords:** *Clostridioides difficile*, colitis, COVID-19, diarrhea, infection, pandemic

## Abstract

The COVID-19 pandemic has changed the way we practice medicine and lead our lives. In addition to pulmonary symptoms; COVID-19 as a syndrome has multisystemic involvement including frequent gastrointestinal symptoms such as diarrhea. Due to microbiome alterations with COVID-19 and frequent antibiotic exposure, COVID-19 can be complicated by *Clostridioides difficile* infection. Co-infection with these two can be associated with a high risk of complications. Infection control measures in hospitals is enhanced due to the COVID-19 pandemic which in turn appears to reduce the incidence of hospital-acquired infections such as *C. difficile* infection. Another implication of COVID-19 and its potential transmissibility by stool is microbiome-based therapies. Potential stool donors should be screened COVID-19 symptoms and be tested for COVID-19.

## Tweetable abstract

#*Clostridioides difficile* complicating #COVID-19 can be associated with high mortality. COVID-19 patients with diarrhea, especially those who received antibiotics recently should be tested for *C. difficile*.

Almost all aspects of medicine have been altered by the emergency of the COVID-19 pandemic caused by the SARS-CoV-2. These include but not limited to the timing of elective procedures and surgeries, how and when we provide preventative healthcare, patient willingness to present for emergency care and the collateral impact on other illnesses, some of which would be comorbidities that worsen with SARS-CoV-2 infection or its treatment [1].

As of 25 January 2021, according to the Johns Hopkins COVID-19 tracker; there have been over 99 million COVID-19 cases reported worldwide and it is apparent that COVID-19 was now the most common infection seen in hospitals in 2020 and in 2021. An infection caused by the SARS-CoV-2 can range from mild symptoms to multi-organ involvement leading to death with a few known predictors of adverse outcomes [2]. Some humans remain asymptomatic carriers but may still transmit and lead to infections in those vulnerable to COVID-19 [3]. Prior to the COVID-19 pandemic, the most common organism causing infections in hospitals in the USA was *Clostridioides difficile* infection (CDI), and has been classified as an urgent threat by the US Centers for Disease Control [4]. In this article, we report the interplay of COVID-19 with initial and recurrent CDI: highlighting risks of CDI, infection control practices and how COVID-19 is impacting the treatment of CDI as it relates to microbiome-based therapies.

## Incidence & risks of CDI with COVID-19

With the advent of COVID-19, came several challenges one of which was therapeutics and several experimental therapeutics have been tried [5]. These have included antibiotics such as azithromycin among antivirals, and other therapies [6]. With severe pulmonary involvement, broad-spectrum antibiotics are being used for superimposed or suspected bacterial infection. The risk factors for CDI include age, antibiotic exposure, hospitalization and comorbid conditions. When patients with COVID-19, receive antibiotics they are at a risk of antibiotic-associated diarrhea and CDI. These risk factors are associated with dysbiosis of the microbiome leading to CDI. There have been recent reports of COVID-19 causing microbial dysbiosis in the gut similar to antibiotic exposure and as seen in patients with recurrent CDI [7]. Additionally, to complicate the clinical picture, SARS-CoV-2 frequently leads to gastrointestinal symptoms including nausea, vomiting and diarrhea owing to the extensive presence of the angiotensin converting enzyme II in the gut [8]. A common symptom of COVID-19 and CDI is diarrhea which can complicate the diagnosis of this co-infection owing to lack of clinical suspicion when one of these infections is initially diagnosed.

An interesting retrospective cohort analysis from New York, NY compared a pre-COVID-19 cohort of all adult patients diagnosed with CDI with a cohort during the COVID-19 pandemic [9]. Overall, hospital onset standardized infection ratios were not different in hospital-onset CDI rate despite a trend toward increased high-risk antibiotic exposure. There was a trend toward an increased length of stay and a trend toward decreased *C. difficile* testing volumes, suggesting that patients’ diarrhea and gastrointestinal symptoms could have been attributed to COVID-19 [9]. A possible explanation could be increased and enhanced infection control measures seen in healthcare facilities during the COVID-19 pandemic. A study from Madrid, Spain assessed the effect of COVID-19 on nosocomial CDI incidence [10]. Of the 2337 patients admitted with COVID-19, the incidence of healthcare facility acquired CDI was 2.68 per 10,000 patient days (12 patients), significantly lower compared with an incidence of 8.54 per 10,000 patient days, despite a higher overall antibiotic consumption during the COVID-19 pandemic. These data suggested a 70% reduction in CDI incidence suggesting infection control measures at the cornerstone of reducing CDI incidence [10].

A case series of nine patients from Detroit with SARS-CoV-2 and *C. difficile* co-infection included mostly elderly females [11]. The rate of CDI at the center slightly increased from 3.32 to 3.6 per 10,000 patient-days from January to February 2020 until from March to April 2020. A third of these patients had a prior history of CDI and all had a positive nucleic acid amplification assay for *C. difficile* and had diarrhea. All patients were severely ill and had multiple comorbid conditions. The majority of these patients had prior or postadmission antibiotic exposure as precipitating factors for CDI. In these patients, CDI was managed with guideline-based therapies. Of note, one patient received fecal microbiota transplantation (FMT) and one did not receive antibiotics and was considered to be colonized with *C. difficile*. The overall mortality was 44.4% (n = 4) and one was discharged to hospice [11]. The mortality is not attributed to *C. difficile* and SARS-CoV-2 co-infection and the high mortality rate is confounded by age and comorbid conditions. There is an overall paucity of outcomes data in patients with these co-infections. This high mortality, however, remains concerning and suggests that modifiable risk factors such as antibiotics should be judiciously used in patients with COVID-19. Antibiotics are life-saving drugs and have been shown to increase human life expectancy. Indicated antibiotics should not be withheld due to fear of developing CDI or other complications. During this pandemic (and otherwise), hospitalized adults with unexplained diarrhea and outpatients with unexplained diarrhea in the presence of risk factors should be tested for the presence of *C. difficile*.

## Infection control practices

An important aspect to prevent the spread of CDI and COVID-19 is the practice of infection control [12]. While taking care of a patient with COVID-19, contact and droplet precautions are recommended. Additionally, while performing aerosol-generating procedures in patients with COVID-19, a respirator such as a powered air purifying respirator or an N95 mask with a face shield is recommended. As we are in a pandemic, respiratory precautions with masks are being recommended for regular patient care and for social interactions. This had initially led to a shortage of personal protective equipment (PPE) which still continues in the US and several parts of the world [13]. It is known that CDI spreads from person to person and adequate contact precautions and hand washing can limit its spread [14]. Asymptomatic colonizers are typically not tested or subject to isolation. While we employ hygiene measures to prevent the spread of COVID-19, it is conceivable that the spread of infections such as CDI will be curbed due to these measures. On the other hand, if the supply of PPE is depleted in a healthcare setting forcing providers to reuse PPE, it may augment the spread of infections such as CDI.

## Microbiome-based therapies & COVID-19

There is considerable evidence that CDI is associated with high rates of recurrence and evidence-based recommendations suggest the use of microbiome-based therapies such as FMT for prevention of future CDI recurrences [15]. For the last decade, FMT has been a part of mainstream medicine to successfully alleviate recurrent CDI. The components of stool are not well defined, but consist of water, bile acids, bacteria, viruses, fungi, human colonic cells and undigested food. The bacteria, viruses and fungi comprise the gut microbiota with their collective genome known as the microbiome [16]. Microbial dysbiosis clearly has a cause–effect relationship with recurrent CDI and correction of this dysbiosis with FMT resolves CDI. FMT is dependent on procurement of stool from healthy well-screened donors under the guidance of the US FDA.

Despite the publication of case series, cohort studies and clinical trials, outlining donor screening and methodological components of FMT; there have been instances of bacterial infection transmission with FMT [17]. It has been determined that SARS-CoV-2 can be transmitted from intimate or close person-to-person contact, fomite spread, airborne spread and fecal–oral spread due to the presence and isolation of SARS-CoV-2 from stool and sewage samples [18]. It is also known that SARS-CoV-2 can be present in stool despite being absence from respiratory samples [19]. These confirmed and possible transmissions have implications for donor screening and performance of FMT. In March 2020, the FDA issued a guidance of caution for performance of FMT and the need for screening donors for SARS-CoV-2 prior to performing FMT [20]. So far, no cases of SARS-CoV-2 transmission have been described with FMT.

In patients with suspected recurrent CDI, the first step would be to delineate a true recurrence from postinfection irritable bowel syndrome or alternate causes of diarrhea. Frequently, patients with history of CDI may remain colonized with *C. difficile* and test positive for *C. difficile* on a stool test. In order to make an accurate diagnosis of CDI would defined by the presence of risk factors for CDI, presence of symptoms suggestive of CDI (three or more loose or watery stools a day), positive stool assay for *C. difficile* and a response to antibiotic treatment for CDI within 3–4 days of starting antibiotics [21]. After successful resolution of CDI with antibiotics, patients tend to have a recurrence within 8 weeks of stopping antibiotic therapy. In patients with truly multiply recurrent CDI, the first step is to initiate an antibiotic therapy and then discuss a microbiome-based therapy such as FMT.

Donor screening and testing needs to evolve with the COVID-19 pandemic [21]. As of the writing of this article, OpenBiome (the largest stool bank in North America) has ceased operations under the direction of the FDA. If a person is being considered to be a stool donor for performing an FMT, the first step would be to perform a health screen for microbiome alternation-associated diseases and screening for transmissible infection. Similar to other viruses, there is an incubation period for COVID-19, when one is asymptomatic but can be transmitting virus to another person [21]. Screening for COVID-19 would begin prior to donation. Screening for COVID-19 at minimum would include exposure screening, symptom screening and a nucleic acid-based assay with a nasal or nasopharyngeal swab [21]. Any positive result would disqualify from being a stool donor. For donations being frozen, exposure and symptom screening should be repeated at every donation. In a stool bank model, release screening where testing should be performed every 2 weeks, with stool samples collected in between be kept frozen and quarantined for use. If the follow-up testing is negative; the doses may be released and used for FMT. This release screening at minimum should include a nucleic acid-based assay and potentially serology testing with IgG to evaluate for interim seroconversion [21]. For donations being used fresh, donor testing should be performed no earlier than 48 h prior to donation and donors should be requested to avoid all exposures to COVID-19. There is a small risk of false-negative tests. Release screening (with nucleic acid assay and serology) after quarantining of stool samples will help mitigate this risk. In an ideal world, a validated and rapid stool test for SARS-CoV-2 will simplify donor screening, but those tests are not readily available at this time.

## Conclusion

COVID-19 is a multisystemic illness with frequent gastrointestinal symptoms such as diarrhea with studies implicating an altered gut microbiome in patients with COVID-19. Enhanced infection control measures due to the pandemic appears to be reducing hospital-acquired infections. Hospitalized COVID-19 patients are at a risk for developing CDI and this co-infection can be associated with a higher rate of complications. Since the SARS-CoV-2 virus can be present in stool even in asymptomatic individuals, there is potential transmissibility by stool. Potential stool donors enrolled for microbiome restoration programs should be screened COVID-19 symptoms and be tested for COVID-19.

## Future perspective

As we understand COVID-19 therapeutics and develop preventive measures such as vaccines and enhanced public health infection control measures; there will be a lesser reliance on antibiotics for management of COVID-19. Early recognition of pulmonary complications and prompt management with disease course-altering medications such as antivirals or steroids could prevent the use of antibiotics and avoid co-infection with *C. difficile*. It will be interesting to study if the gut microbiome alterations seen in SARS-CoV-2 predispose patients to CDI. Additionally, the association of gut microbiome alterations in SARS-CoV-2 with disease severity is being explored. It would be interesting to study microbiota restoration therapies to modulate the microbiome to impact disease severity. It is imperative to prevent this co-infection due to high impending mortality in these patients. With *C. difficile* being the commonest hospital bacterial infection and SARS-CoV-2 the commonest viral infection, the relationship between these two infections needs to be further studied. Lack of reporting of these patients owing to a pandemic situation is a likely explanation of paucity of data. Outcomes in patients with this co-infection should be elucidated in a larger cohort controlling for confounding variables such as age and comorbid conditions. Development of validated stool assays for SARS-CoV-2 would help detect gastrointestinal involvement and prevent disease spread.

**Executive summary**

**Incidence & risks of CDI with COVID-19**

- COVID-19 frequently presents with gastrointestinal symptoms such as diarrhea, nausea and vomiting.
- *Clostridioides difficile* infection may complicate COVID-19 and lead to high mortality.

**Infection control practices**

- Enhanced infection control measures for COVID-19 seem to be decreasing the incidence of hospital-acquired *C. difficile* infection.

**Microbiome-based therapies & COVID-19**

- Pandemics like COVID-19 complicate the use of therapeutics such as fecal microbiota transplantation for *C. difficile* infection which are dependent on procurement of stool from healthy donors.
- Validated stool assays for COVID-19 are urgently needed.
